# Cannabidiol (CBD) modulates the serotonin–kynurenine metabolic balance in the brain of morphine-tolerant neuropathic rats

**DOI:** 10.1007/s43440-026-00850-w

**Published:** 2026-03-24

**Authors:** Sofia Nasini, Leora Pearl-Dowler, Martha Lopez-Canul, Luca Posa, Antonella Bertazzo, Gabriella Gobbi, Stefano Comai

**Affiliations:** 1 Department of Pharmaceutical and Pharmacological Sciences, University of Padua, Largo Meneghetti 2, 35131 Padua (PD), Italy; 2 Department of Psychiatry, McGill University, Montreal, QC Canada; 3 Research Institute, McGill University Health Center, McGill University, Montreal, QC Canada; 4 Department of Biomedical Sciences, University of Padua, Padua, Italy

**Keywords:** Morphine tolerance, Neuropathic pain, Cannabidiol, Tryptophan metabolism, Dopamine

## Abstract

**Background:**

Neuropathic pain (NP) is a chronic and debilitating condition that remains difficult to treat, with opioids like morphine often leading to tolerance and reduced efficacy over time. Cannabidiol (CBD) has emerged as a potential therapeutic for NP even in morphine tolerance conditions; however, its effects on nociceptive modulation and neurotransmitter metabolism in morphine-tolerant NP models remain poorly understood. This study investigated the impact of CBD on mechanical hypersensitivity and on tryptophan (TRP) metabolism via the serotonin (5-HT) and kynurenine (KYN) pathways and dopamine (DA) in Wistar rats with NP and morphine tolerance.

**Methods:**

NP was induced using the spared nerve injury (SNI) model. Animals received morphine (5 mg/kg, subcutaneous (sc), twice daily) for 7 days to induce tolerance. On Day 8, rats were treated with CBD (20 mg/kg, ip) or vehicle. Mechanical hypersensitivity was assessed, and TRP metabolism and DA were evaluated in different brain regions and serum.

**Results:**

Repeated morphine induced tolerance to its analgesic effects. CBD significantly reduced mechanical hypersensitivity in non-tolerant rats, with a modest effect in morphine-tolerant animals. In tolerant rats, CBD increased 5-hydroxytryptophan (5-HTP)/TRP ratio in hippocampus and midbrain and reduced KYN levels in the hippocampus, indicating a shift toward 5-HT synthesis. CBD also lowered DA and the 5-HTP/TRP ratio in the brainstem, and decreased serum TRP and 5-HT.

**Conclusions:**

CBD treatment in NP morphine-tolerant rats exerted a modest effect on mechanical hypersensitivity and was associated with a partial counteraction of metabolic dysregulation by reducing KYN production and shifting TRP metabolism toward 5-HT synthesis (increased 5-HTP/TRP ratio). These findings suggest that CBD modulates 5-HT tone through region-specific mechanisms associated with the development of morphine tolerance.

## Introduction

Neuropathic pain (NP) is a chronic disorder arising from injury or dysfunction of the peripheral or central nervous system, leading to persistent alterations in somatosensory processing [1]. The prevalence of NP in the general population ranges from 6 to 8% [2], accounting for approximately 20–25% of all chronic pain conditions [3]. NP is characterized by spontaneous and evoked pain, including mechanical hypersensitivity (allodynia) and hyperalgesia [4], as well as paraesthesia and dysesthesia [4, 5]. Patients often describe burning, shooting, or stabbing sensations, frequently accompanied by numbness, tingling, and heightened sensitivity to touch [6, 7]. Clinical guidelines recommend non-opioid pharmacological treatments such as tricyclic antidepressants, serotonin-norepinephrine reuptake inhibitors, anticonvulsants, and topical agents as first-line therapies [8]. These agents primarily target neurotransmitter systems, including serotonin (5-HT) and dopamine (DA), which modulate pain transmission [9]. Nonetheless, opioids are frequently prescribed as second-line treatments due to historical use, patient demand, and limited efficacy of first-line drugs. Chronic opioid therapy, however, is hampered by tolerance, dependence, and addiction [10]. Once tolerance develops, strategies to maintain analgesic efficacy remain limited. Cannabidiol (CBD), the major non-psychoactive constituent of cannabis, has emerged as a candidate for NP management. Preclinical and clinical studies suggest that CBD engages anti-inflammatory, antioxidant, and neuroprotective mechanisms [11]. Importantly, CBD inhibits indoleamine 2,3-dioxygenase (IDO) [12], the rate-limiting enzyme converting tryptophan (TRP) into kynurenine (KYN), which is upregulated in neuroinflammatory and neurodegenerative states [13]. Inhibition of IDO may shift TRP metabolism toward the 5-HT biosynthetic pathway, promoting 5-HT neurotransmission, a key modulator of nociception and affective states [14]. 5-HT is synthesized from TRP in two enzymatic steps: hydroxylation by tryptophan hydroxylase (TPH) to 5-hydroxytryptophan (5-HTP), followed by decarboxylation via aromatic L-amino acid decarboxylase to 5-HT [13]. Of the two TPH isoforms, TPH1 is mainly expressed peripherally, whereas TPH2 is localized in the CNS [15] and drives neuronal 5-HT synthesis. CBD also reduces pro-inflammatory cytokines such as interferon-gamma (IFN-γ) and tumor necrosis factor-alpha (TNF-α), both of which activate the KYN pathway [16, 17].

Dysregulated TRP metabolism has been linked to NP pathophysiology. Conversion of TRP to KYN is catalyzed by tryptophan 2,3-dioxygenase (TDO) and indoleamine 2,3-dioxygenases 1 and 2 (IDO1, IDO2), with IDO isoforms contributing prominently under pathological conditions [13]. Altered KYN pathway activity results in imbalanced production of downstream metabolites with neurotoxic or neuroprotective effects [18, 19].

Beyond TRP metabolism, CBD modulates dopaminergic function, including DA release, reuptake, and receptor signaling, which may further contribute to its analgesic profile [20]. However, the extent to which CBD regulates KYN and 5-HT pathways, and its interaction with DA signaling in NP, remains poorly understood.

In this study, we thus investigated whether CBD can alleviate neuropathic pain-related behaviour in morphine-tolerant rats and counteract the monoaminergic alterations associated with opioid tolerance. Using the Spared Nerve Injury (SNI) model of neuropathic pain, CBD was administered after tolerance to morphine had been established. To explore potential neurochemical mechanisms, TRP metabolism was analyzed in brain regions implicated in pain processing, the prefrontal cortex (PFC), hippocampus, midbrain, and brainstem, as well as in serum. Specific metabolites quantified included TRP, 5-HTP, 5-HT, 5-hydroxyindoleacetic acid (5-HIAA), and KYN. Additionally, dopamine (DA) levels were measured, given its key role in pain modulation and its dysregulation during chronic opioid exposure.

## Materials and methods

### Experimental design

**Fig. 1:**
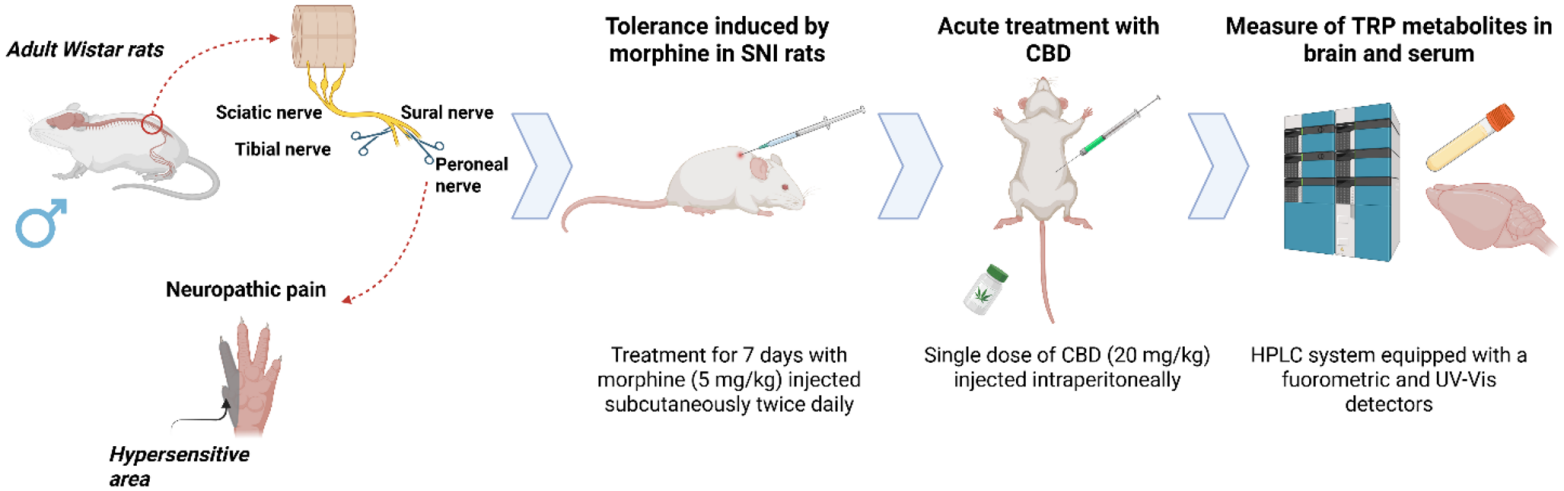
The study was conducted using adult male wistar rats. Neuropathic pain was induced via the spared nerve injury (SNI) model, where the tibial and peroneal branches of the sciatic nnerve were severed, leaving the sural nerve intact. Following recovery, morphine tolerance was induced by administering morphine (5 mg/kg, sc) twice daily for 7 days. On day 8, rats received an acute injection of cannabidiol (CBD, 20 mg/kg, Ip). Finally, brain regions and serum were collected for biochemical analysis, where tryptophan (TRP) and its downstream metabolites were quantified using high-performance liquid chromatography (HPLC) equipped with fluorometric and UV-VIS detectors

NP was induced using the SNI model described by Decosterd and Woolf [21], in which the tibial and peroneal nerves were severed while leaving the sural nerve intact. Following recovery, naïve (with intact nerve) and NP (with lesioned nerve) rats were treated sc with morphine (5 mg/kg, *n* = 12 rats) or vehicle (0.9% NaCl, *n* = 13 rats) twice daily for 7 days. Mechanical hypersensitivity was assessed using von Frey filaments at baseline, and 30 min post-treatment on days 1 and 7. Morphine-tolerant and non-tolerant rats received a single dose of cannabidiol (CBD, 20 mg/kg [22], intraperitoneal (Ip); *n* = 6 for morphine-tolerant rats and *n* = 6 for morphine-non-tolerant rats) or vehicle (*n* = 6 for morphine-tolerant rats and *n* = 7 for morphine-non-tolerant rats) and tested for mechanical hypersensitivity 30 min, 1, 2, 3, 4, and 5 hours post-treatment. Then, several brain regions, including the prefrontal cortex (PFC), hippocampus, midbrain, and brainstem, along with the blood, were collected for the quantification of TRP and its metabolites, and DA. Figure 1 illustrates the experimental timeline.

### Animals

Male Wistar rats (Charles Rivers, Quebec, Canada), weighing 130 g at the start of the experiments, were housed in groups of 2–3 per cage in a temperature and humidity-controlled facility with a 12:12 h light-dark cycle (lights on at 7 AM) and provided with ad libitum access to food and water. All experimental protocols were carried out during the light phase (7 am-7 pm) and approved by the McGill University Animal Ethics Committee (Protocol # 7181), in accordance with the IASP ethical guidelines for the investigation of experimental pain in conscious animals [23] and the Canadian Institutes of Health Research guidelines for animal care and experimental use. For all behavioural experiments, the experimenter was blind to drug treatment. The number of rats per experimental group was based on our previous research [24, 25]. To minimize cage effects and environmental biases, animals were selected one at a time from different cages in a randomized manner for each experimental session. NP rats were monitored daily by the McGill Comparative Medicine and Animal Resources Centre (CMAR). Animals exhibiting signs of distress or suffering were treated or euthanized in accordance with the institutionally approved Standard Operating Procedures for humane endpoints (SOP #410).

### Drugs

Morphine sulphate (Sigma Aldrich, St Louis, MO; Cayman Chemical, Ann Arbor, MI) was dissolved in saline (0.9% NaCl) and injected sc at the dose of 5 mg/kg twice per day for 7 days. Cannabidiol (CBD) (Cayman Chemical, Ann Arbor, MI) was dissolved in a vehicle composed of EtOH, Tween80, and saline (0.9% NaCl) in a ratio of 3:1:16 and administered ip at a single dose of 20 mg/kg for behavioural experiments. Solutions for injections were prepared by a designated experimenter, while Ip injections were performed in a blinded fashion by independent investigators (MLC or LPD).

### Spared nerve injury (SNI)

The SNI procedure was performed following Decosterd and Woolf (21). Rats were anesthetized with 5% isoflurane in oxygen and maintained with 2–3% isoflurane during surgery, with the absence of a toe-pinch reflex confirming full anesthesia. An ophthalmic ointment was applied before starting surgery to protect the eyes. The right hind limb was shaved, and an incision was made to expose the sciatic nerve. The common peroneal and tibial branches of the nerve were ligated with 4.0 mm vicryl suture and transected, leaving the sural nerve intact. The muscle layer was sutured with 3.0 vicryl, and the skin was closed with EZclips. Carprofen (5 mg/kg, sc) was administered preoperatively and for 2 days postoperatively for analgesia. Wound closures were removed 10 days after surgery.

### Assessment of mechanical hypersensitivity

Fourteen days after SNI, rats weighing approximately 300 g were tested for mechanical hypersensitivity using von Frey filaments through the up-down method described by Chaplan et al. [26].

Briefly, rats (*n* = 12–13/group) were placed in individual plexiglass cubicles on a wire mesh surface and allowed to habituate for 45 min. First, a filament exerting a standard force of 4.31 grams was applied to the plantar surface of the right hind paw, ipsilateral to the nerve injury, for 10 s. If the stimulus elicited a withdrawal of the paw, a lower force was applied; if no response occurred, a higher force was used, up to a maximum of 15 g.

The 50% Paw withdrawal threshold (PWT) was calculated using the equation proposed by Dixon [27]. Rats with a baseline PWT < 4 g were classified as allodynic and included in the study [26, 28], while the two rats displaying motor impairments (e.g., a visible limp, foot drop, or paw dragging) were excluded. Behavioral assessments of mechanical hypersensitivity using Von Frey filaments were performed in a blinded fashion by independent investigators (MLC or LPD).

### Euthanasia and tissue preparation

Six hours after the injection of CBD or vehicle, rats were anesthetized with 5% isoflurane in oxygen until full anesthesia was achieved, then euthanized by decapitation. Blood was collected from the trunk, left at room temperature for 10–15 min to clot, centrifuged at 4 °C and 2000 rpm for 10 min, and the serum was collected and stored at −80 °C until the analysis. The brain was rapidly dissected, and the PFC, hippocampus, midbrain, and brainstem were flash frozen in dry ice and stored at −80 °C until the analyses.

### Quantification of Trp and its degradation into 5-HT and Kyn

Quantitative analysis of TRP, 5-HTP, 5-HT, and its metabolite 5-HIAA, KYN, and DA, was performed in the PFC, hippocampus, brainstem, and midbrain of SNI rats tolerant and nontolerant to morphine and treated with vehicle or CBD (VEH *n* = 10–14, CBD *n* = 10–14) using a well-validated HPLC method in our laboratory [24]. Briefly, brain regions were homogenized and sonicated for 2 min in 200 μL of a 0.2 M perchloric acid solution and then centrifuged for 6 min at 13.000 g at 4 °C. In serum samples, protein precipitation was conducted by adding 100 µl of acetonitrile to 100 µl of serum; the samples were then centrifuged for 6 min at 13.000 g and the supernatant collected for the analysis of TRP, 5-HTP, 5-HT, and KYN. Separation and then quantification of TRP, 5-HTP, 5-HT, 5-HIAA, and DA were carried out with a Shimadzu LC-10AD HPLC system equipped with a Shimadzu RF-10AXL fluorometric detector set at excitation and emission wavelengths of 279 and 320 nm, respectively. Chromatographic separation was performed on an Apollo C18 (5 μm; 250 mm ×4.6 mm) column (Sepachrom Mega Srl, Milan, IT) using a mobile phase at a flow rate of 1 mL/min composed of milliQ water/acetonitrile (5% water, 95% acetonitrile) and milliQ water/methanol (90% water, 10% methanol) in a 5:95 v/v, respectively, that were acidified with orthophosphoric acid to a pH of 3.5. KYN was determined using a Shimadzu LC-10AD HPLC system equipped with a Shimadzu SPD-10A UV-VIS detector set at 360 nm, a Robusta C18 (5 μm; 250 mm ×4.6 mm) column (Sepachrom Mega Srl, Milano, IT), and a mobile phase as indicated above for TRP metabolites via 5-HT, at a flow rate of 1 ml/min. The KYN/TRP and 5-HTP/TRP ratios were calculated as an indirect measure of the activity of the IDO and tryptophan 5-hydroxylase (TPH) enzymes, respectively [29, 30]. Biomarker analysis and quantification were conducted in blinded conditions by S.N., A.B. and S.C.

### Statistical analysis

Statistical analyses were performed using GraphPad Prism Version 8.0.0 for Windows (GraphPad Software, San Diego, CA, USA). The Shapiro-Wilk normality test was used to assess the normality of the data. Three-way ANOVA followed by the Bonferroni multiple comparison post hoc test was used to evaluate the effects of morphine tolerance, CBD treatment, and time post CBD treatment on mechanical hypersensitivity. For biochemical data, a two-way ANOVA was performed to evaluate the main effects of morphine tolerance and CBD treatment. When significant interactions or main effects were found, Bonferroni’s multiple comparison post hoc test was applied. Post hoc comparisons were performed for all simple main effects to comprehensively assess differences between all group combinations and evaluate the restoration to non-tolerant levels. The significance level was set at *p* < 0.05. Data are expressed as mean ± standard error of the mean (SEM).

## Results

### Modest analgesic effects of CBD in morphine-tolerant NP rats

**Fig. 2:**
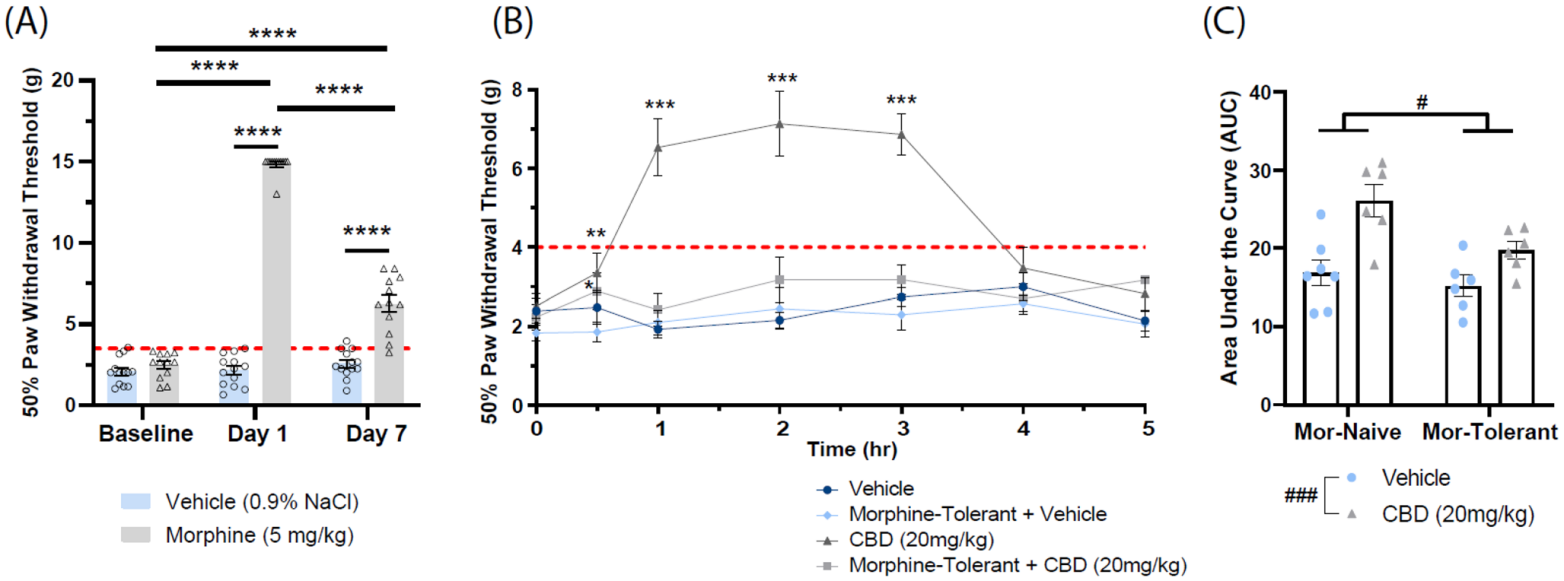
Cannabidiol (CBD) alleviates mechanical hypersensitivity in sciatic nerved injured (SNI) rats. (**A**) Repeated morphine administration (5 mg/kg, sc, twice daily for 7 days) significantly reduced mechanical hypersensitivity on Day 1, followed by a loss of efficacy by Day 7, confirming the development of morphine tolerance in SNI rats. (**B**) Time course of paw withdrawal threshold (PWT) following a single administration of CBD (20 mg/kg, ip) in morphine-tolerant and non-tolerant rats. Three-way ANOVA revealed significant main effects of time (*p* < 0.0001), tolerance (*p* = 0.0004), and CBD treatment (*p* < 0.0001), as well as significant interactions (time × tolerance, time × CBD treatment, and tolerance × CBD treatment), indicating that the effect of CBD on mechanical hypersensitivity was modulated by morphine tolerance. (**C**) Area under the curve (AUC) analysis of the PWT over the 5-hour testing period revealed significant main effects of morphine tolerance and CBD treatment (***p < 0.001), without a significant interaction. Data are presented as mean ± SEM (*N* = 5–7 rats per group). *p < 0.05, **p < 0.01, ***p < 0.001, ****p < 0.0001, two-way ANOVA for repeated measures followed by Bonferroni post hoc test. # *p* < 0.05, ###*p* < 0.001, main effect of morphine tolerance or CBD treatment following two-way ANOVA

The effects of morphine tolerance and CBD treatment on mechanical hypersensitivity, assessed as a measure of NP-related responses, are presented in Fig. 2. Repeated morphine administration over seven days induced tolerance to its analgesic effects in NP rats. This was evidenced by a significant time ×morphine interaction (F _2, 46_ = 340.2, *p* < 0.0001) and a significant main effect of morphine (F _1, 23_ = 353.2, *p* < 0.0001) and time (F _1.937, 44.56_ = 335.1, *p* < 0.0001). Post hoc Bonferroni comparisons confirmed that mechanical hypersensitivity was significantly attenuated on Day 1 (D1) of morphine treatment compared to vehicle-treated rats. However, by Day 7, morphine-treated rats exhibited a significant decrease in the paw withdrawal threshold (PWT) relative to Day 1, indicating the development of morphine tolerance. In contrast, vehicle-treated rats did not show significant changes in PWT over time.

As shown in Fig. 2B–C, CBD (20 mg/kg) significantly modulated mechanical hypersensitivity in NP-like behaviour of rats, with differential effects depending on morphine tolerance. Three-way ANOVA revealed significant main effects of time (F _6, 126_ = 11.66, *p* < 0.0001), morphine tolerance (F _1, 21_ = 17.49, *p* = 0.0004), and CBD treatment (F _1, 21_ = 34.36, *p* < 0.0001). Significant interactions were also found between time and tolerance (F _6, 126_ = 6.612, *p* < 0.0001), time and CBD (F _6, 126_ = 10.34, *p* < 0.0001), tolerance and CBD (F _1, 21_ = 10.42, *p* = 0.0040), as well as between time, tolerance, and CBD (F _6, 126_ = 10.18, *p* < 0.0001), indicating that the mechanical hypersensitivityresponse to CBD varies over time and is influenced by prior repeated morphine exposure. However, although CBD administration produces a modest increase in PWT thresholds in morphine-tolerant SNI rats, these effects remain below the established anti-allodynic threshold, represented by the red dashed line in Fig. 2B.

To further quantify the overall effect during the 5-hour follow-up, the analysis of the area under the curve (AUC) was performed. Two-way ANOVA of AUC (Fig. 2C) confirmed significant main effects of morphine tolerance (F _1, 21_ = 6.211, *p* = 0.0211) and CBD treatment (F _1, 21_ = 18.67, *p* = 0.0003), although no significant interaction between the two factors was observed (F _1, 21_ = 2.111, *p* = 0.1610).

### Prefrontal cortex (PFC)

**Fig. 3:**
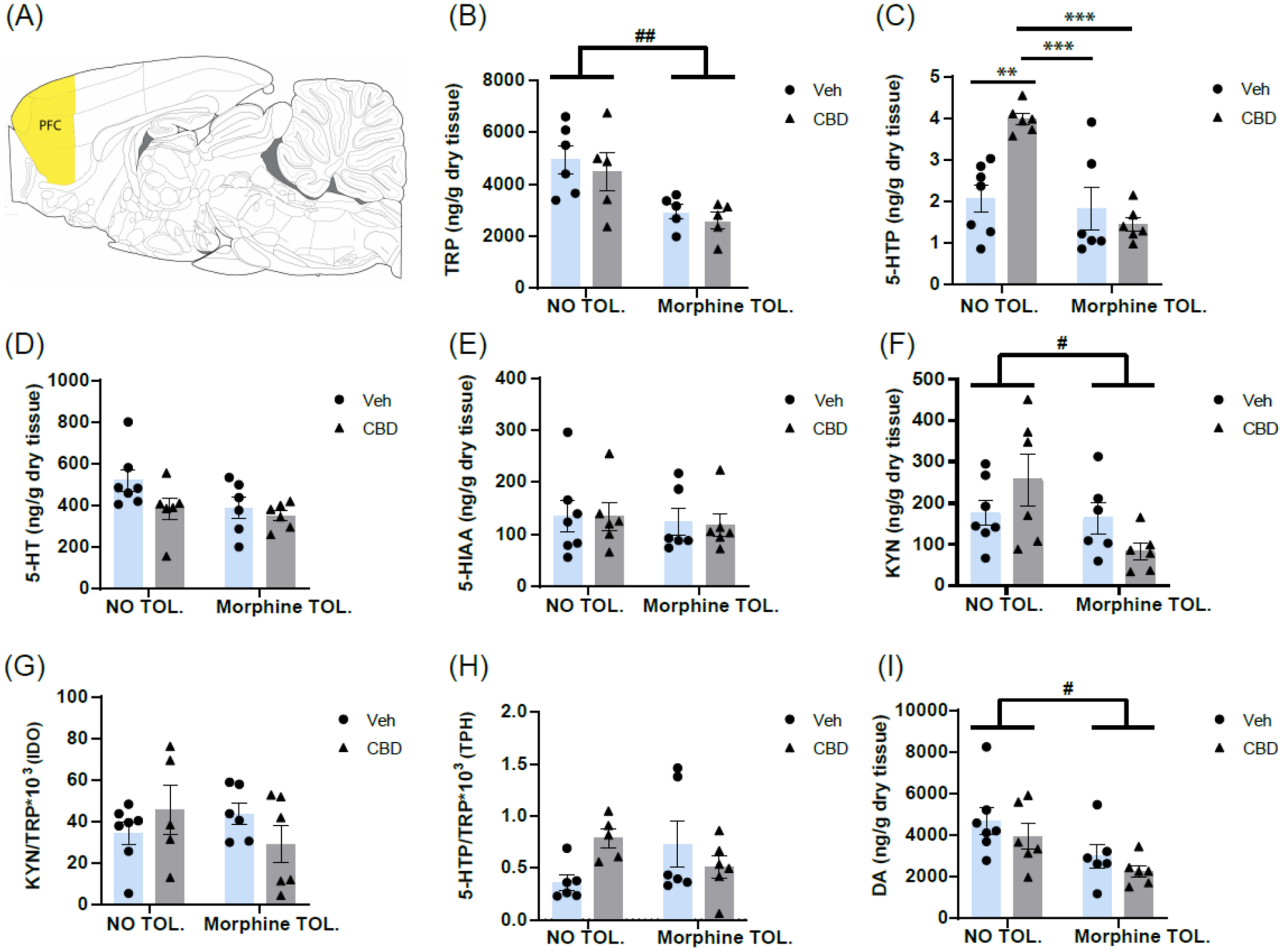
Effects of cannabidiol (CBD) treatment in sciatic nerve injured (SNI) rats tolerant (morphine TOL.) and non-tolerant (NO TOL.) to morphine on the metabolites of the tryptophan to serotonin and kynurenine pathways and dopamine in the prefrontal cortex (PFC): panels show (**a**) Sagittal section of PFC rat brain (Coordinates: Interaural 11.76 mm, Bregma 2.76 mm), from Paxinos and Watson [31], PFC levels of (**b**) Tryptophan (TRP), (**c**) 5-hydroxytryptophan (5-HTP), (**d**) 5-hydroxytryptamine (5-HT), (**e**) 5-hydroxyindole acetic acid (5-HIAA), (**f**) Kynurenine (KYN), (**g**) 5-HTP/TRP*1000 ratio as a proxy of the activity of the enzyme tryptophan 5-hydroxylase (TPH), (**h**) KYN/TRP*1000 ratio as a proxy of the activity of the enzyme indoleamine 2,3-dioxygenase (IDO), and (**i**) Dopamine (DA). Data are presented as mean ± SEM (*N* = 5–7 rats per group) with individual values indicated by dots (vehicle, veh) or triangles (CBD). **p < 0.01 and ***p < 0.001, two-way ANOVA followed by Bonferroni post hoc test. # *p* < 0.05 and ## *p* < 0.01 main effect of morphine tolerance following two-way ANOVA

The levels of various metabolites in the PFC of SNI rats tolerant to morphine and treated with CBD are shown in Fig. 3.

SNI rats that developed tolerance to morphine exhibited significantly lower PFC levels of TRP compared to non-tolerant rats, regardless of CBD treatment (effect of morphine tolerance: F _1, 17_ = 13.8, *p* = 0.0016; effect of CBD treatment: F _1, 17_ = 0.60, *p* = 0.4467; interaction morphine tolerance ×CBD treatment: F _1, 17_ = 0.011, *p* = 0.9188; Fig. 3B). Concerning PFC levels of 5-HTP (effect of morphine tolerance: F _1, 21_ = 17.92, *p* = 0.0004; effect of CBD treatment: F _1, 21_ = 5.632, *p* = 0.0273; interaction morphine tolerance ×CBD treatment: F _1, 21_ = 12.56, *p* = 0.0019; Fig. 3C), CBD treatment increased 5-HTP levels in SNI rats not tolerant to morphine, but this effect was not observed in morphine-tolerant rats. Morphine tolerance and CBD treatment had no significant effects on the PFC levels of 5-HT (effect of morphine tolerance: F _1, 21_ = 2.966, *p* = 0.0998; effect of CBD treatment: F _1, 21_ = 3.343, *p* = 0.0817; interaction morphine tolerance ×CBD treatment: F _1, 21_ = 1.027, *p* = 0.3225; Fig. 3D), 5-HIAA (effect of morphine tolerance: F _1, 21_ = 0.2375, *p* = 0.6311; effect of CBD treatment: F _1, 21_ = 0.0121, *p* = 0.9135; interaction morphine tolerance ×CBD treatment: F _1, 21_ = 0.0104, *p* = 0.9199; Fig. 3E), the KYN/TRP*1000 ratio (effect of morphine tolerance: F _1, 21_ = 2.693, *p* = 0.1164; effect of CBD treatment: F _1, 21_ = 0.0411, *p* = 0.8415; interaction morphine tolerance ×CBD treatment: F _1, 21_ = 2.693, *p* = 0.1164; Fig. 3G), or the 5-HTP/TRP*1000 ratio (effect of morphine tolerance: F _1, 21_ = 0.0954, *p* = 0.761; effect of CBD treatment: F _1, 21_ = 0.0559, *p* = 0.4636; interaction morphine tolerance ×CBD treatment: F _1, 21_ = 5.418, *p* = 0.0311; Fig. 3H). Interestingly, PFC KYN levels were significantly lower in morphine-tolerant rats compared to non-tolerant rats, with no detectable effects of CBD (effect of morphine tolerance: F _1, 21_ = 5.387, *p* = 0.0304; effect of CBD treatment: F _1, 21_ = 0.000, *p* = 0.998; interaction morphine tolerance ×CBD treatment: F _1, 21_ = 3.969, *p* = 0.095; Fig. 3F). Finally, PFC DA levels were also reduced in morphine-tolerant rats compared with non-tolerant rats independently of CBD treatment (effect of morphine tolerance: F _1, 21_ = 8,790, *p* = 0.0074; effect of CBD treatment: F _1, 21_ = 1.720, *p* = 0.2039; interaction morphine tolerance ×CBD treatment: F _1, 21_ = 0.000, *p* = 0.9846; Fig. 3I)).

### Hippocampus

**Fig. 4:**
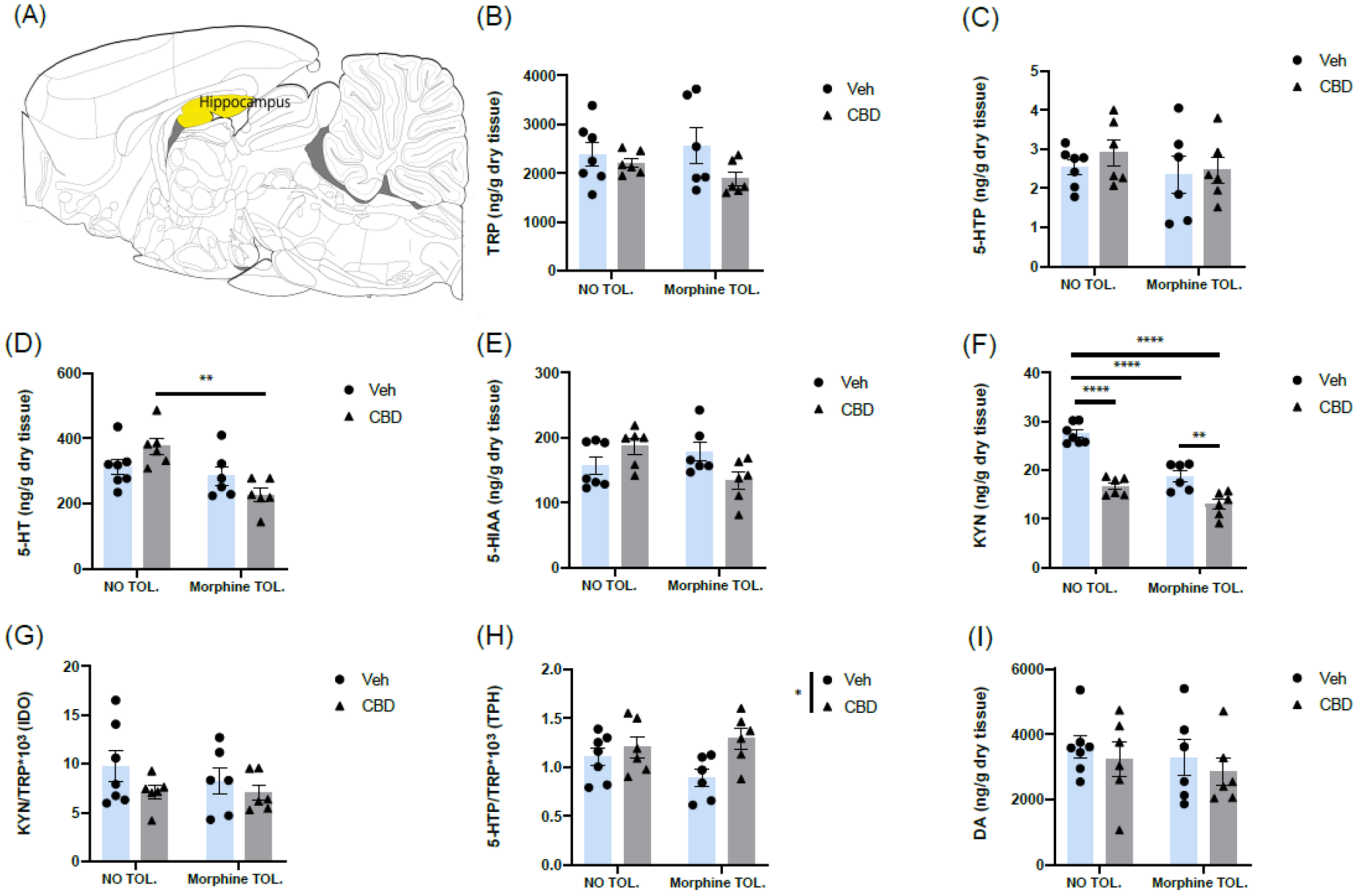
Effects of cannabidiol (CBD) treatment in sciatic nerve injured (SNI) rats tolerant (morphine TOL.) and non-tolerant (NO-TOL.) to morphine on the metabolites of the tryptophan to serotonin and kynurenine pathways and dopamine in the hippocampus: panels show (**a**) Sagittal section of hippocampus rat brain (coordinate: lateral 0.40 mm), from Paxinos and Watson [31], levels of (**b**) Tryptophan (TRP), (**c**) 5-hydroxytryptophan (5-HTP), (**d**) 5-hydroxytryptamine (5-HT), (**e**) 5-hydroxyindole acetic acid (5-HIAA), (**f**) Kynurenine (KYN), (**g**) 5-HTP/TRP*1000 ratio as a proxy of the activity of the enzyme tryptophan 5-hydroxylase (TPH), (**h**) KYN/TRP*1000 ratio as a proxy of the activity of the enzyme indoleamine 2,3-dioxygenase (IDO), and (**i**) Dopamine (DA). Data are presented as mean ± SEM (*N* = 5–7 rats per group) with individual values indicated by dots (vehicle, veh) or triangles (CBD). **p < 0.01 and ****p < 0.0001, two-way ANOVA followed by Bonferroni post hoc test. # *p* < 0.05 main effect of morphine tolerance or CBD treatment following two-way ANOVA

**Fig. 5:**
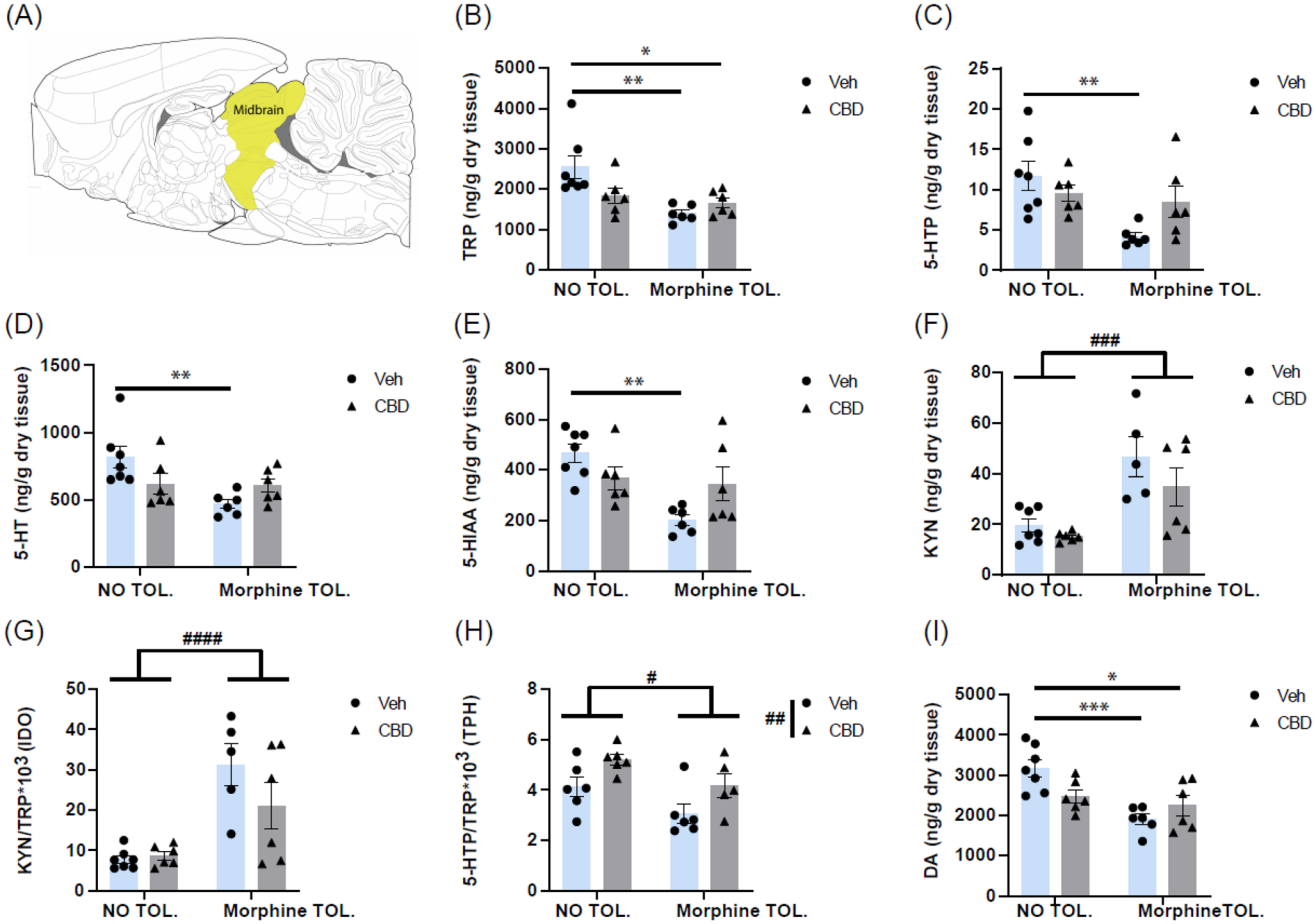
Effects of cannabidiol (CBD) treatment in sciatic nerve injured (SNI) rats tolerant (morphine TOL.) and non-tolerant (NO TOL.) to morphine on the metabolites of the tryptophan to serotonin and kynurenine pathways and dopamine in the midbrain: panels show (**a**) sagittal section of midbrain rat brain (coordinate: lateral 0.40 mm), from Paxinos and Watson [31], levels of (**b**) tryptophan (TRP), (**c**) 5-hydroxytryptophan (5-HTP), (**d**) 5-hydroxytryptamine (5-HT), (**e**) 5-hydroxyindole acetic acid (5-HIAA), (**f**) kynurenine (KYN), (**g**) 5-HTP/TRP*1000 ratio as a proxy of the activity of the enzyme tryptophan 5-hydroxylase (TPH), (**h**) KYN/TRP*1000 ratio as a proxy of the activity of the enzyme indoleamine 2,3-dioxygenase (IDO), and (**i**) dopamine (DA). Data are presented as mean ± SEM (*N* = 5–7 rats per group) with individual values indicated by dots (vehicle, veh) or triangles (CBD). *p < 0.05, **p < 0.01 and ****p < 0.0001, two-way ANOVA followed by Bonferroni post hoc test. # *p* < 0.05, ## *p* < 0.01, ### *p* < 0.001, and #### *p* < 0.0001, main effect of morphine tolerance or CBD treatment following two-way ANOVA

The levels of the various metabolites in the hippocampus of SNI rats tolerant to morphine and treated with CBD are presented in Fig. 4. Unlike in the PFC, the levels of TRP (effect of morphine tolerance: F _1, 21_ = 0.9139, *p* = 0.755; effect of CBD treatment: F _1, 21_ = 0.5290, *p* = 0.0885; interaction morphine tolerance ×CBD treatment: F _1, 21_ = 0.434, *p* = 0.3121; Fig. 4B) and 5-HTP (effect of morphine tolerance: F _1, 21_ = 0.9139, *p* = 0.3500; effect of CBD treatment: F _1, 21_ = 0.5290, *p* = 0.4750; interaction morphine tolerance ×CBD treatment: F _1, 21_ = 0.1434, *p* = 0.7087; Fig. 4C) in the hippocampus were not affected by morphine tolerance or CBD treatment. However, the 5-HTP/TRP*1000 ratio was significantly higher in CDB-treated animals, regardless of morphine tolerance status (effect of morphine tolerance: F _1, 21_ = 0.4292, *p* = 0.5195; effect of CBD treatment: F _1, 21_ = 6.559, *p* = 0.4292; interaction morphine tolerance ×CBD treatment: F _1, 21_ = 2.401, *p* = 0.1362; Fig. 4H).

Morphine-tolerant rats treated with CBD exhibited significantly lower levels of 5-HT (effect of morphine tolerance: F _1, 21_ = 12.44, *p* = 0.0020; effect of CBD treatment: F _1, 21_ = 0.0112, *p* = 0.9166; interaction morphine tolerance ×CBD treatment: F _1, 21_ = 5.979, *p* = 0.0234; Fig. 4D) compared to non-tolerant animals receiving CBD. While an interaction of morphine tolerance ×CBD treatment was detected for hippocampal levels of 5-HIAA, no significant differences were observed among the experimental groups (effect of morphine tolerance: F _1, 21_ = 1.376, *p* = 0.2540; effect of CBD treatment: F _1, 21_ = 0.3214, *p* = 0.5768; interaction morphine tolerance ×CBD treatment: F _1, 21_ = 7.511, *p* = 0.0123; Fig. 4E). CBD treatment significantly reduced the hippocampal levels of KYN in both morphine-tolerant and non-tolerant SNI rats compared with VEH-treated controls (effect of morphine tolerance: F _1, 21_ = 45.00, *p* < 0.0001; effect of CBD treatment: F _1, 21_ = 78.81, *p* < 0.0001; interaction morphine tolerance ×CBD treatment: F _1, 21_ = 7.459, *p* = 0.0125; Fig. 4F)). Furthermore, KYN levels were lower in morphine-tolerant animals treated with both VEH and CBD than in non-tolerant VEH-treated rats. No significant changes were observed in the hippocampal KYN/TRP*1000 ratio (effect of morphine tolerance: F _1, 21_ = 0.3960, *p* = 0.5360; effect of CBD treatment: F _1, 21_ = 2.440, *p* = 0.1332; interaction morphine tolerance ×CBD treatment: F _1, 21_ = 0.3486, *p* = 0.5612; Fig. 4G) or DA levels (effect of morphine tolerance: F _1, 21_ = 0.6275, *p* = 0.4371; effect of CBD treatment: F _1, 21_ = 0.7606, *p* = 0.3930; interaction morphine tolerance ×CBD treatment: F _1, 21_ = 0.000, *p* = 0.9320; Fig. 4I) in response to morphine tolerance or CBD treatment.

### Midbrain

The midbrain comprises key monoaminergic nuclei, including the dorsal raphe nucleus and the ventral tegmental area, which serve as major sources of central serotonin and dopamine, respectively. Given their central role in pain modulation, affective processing, and reward, this region is particularly relevant for evaluating alterations in monoaminergic neurotransmission. The levels of the various metabolites in the midbrain of SNI rats tolerant to morphine and treated with CBD are shown in Fig. 5. Midbrain levels of TRP (effect of morphine tolerance: F _1, 21_ = 10.82, *p* = 0.0035; effect of CBD treatment: F _1, 21_ = 1.218, *p* = 0.2823; interaction morphine tolerance ×CBD treatment: F _1, 21_ = 5.752, *p* = 0.0258; Fig. 5B), 5-HTP (effect of morphine tolerance: F _1, 21_ = 8–337, *p* = 0.0088; effect of CBD treatment: F _1, 21_ = 0.5067, *p* = 0.4844; interaction morphine tolerance ×CBD treatment: F _1, 21_ = 4–753, *p* = 0.0408; Fig. 5C), 5-HT (effect of morphine tolerance: F _1, 21_ = 7.406, *p* = 0.0128; effect of CBD treatment: F _1, 21_ = 0.2067, *p* = 0.6540; interaction morphine tolerance ×CBD treatment: F _1, 21_ = 6.479, *p* = 0.0188; Fig. 5D), and 5-HIAA (effect of morphine tolerance: F _1, 21_ = 10.53, *p* = 0.0039; effect of CBD treatment: F _1, 21_ = 0.2420, *p* = 0.6278; interaction morphine tolerance ×CBD treatment: F _1, 21_ = 7.408, *p* = 0.0128; Fig. 5E) were significantly influenced by the interaction of morphine tolerance ×CBD treatment. Specifically, SNI rats tolerant to morphine and treated with VEH exhibited significantly lower levels of TRP, 5-HTP, 5-HT, and 5-HIAA compared to non-tolerant VEH-treated rats (Fig. 5B–E). Moreover, the levels of TRP were significantly lower in SNI rats tolerant to morphine and treated with CBD compared to non-tolerant rats treated with VEH.

Both KYN levels (effect of morphine tolerance: F _1, 21_ = 20.68, *p* = 0.0002; effect of CBD treatment: F _1, 21_ = 2.484, *p* = 0.1307; interaction morphine tolerance ×CBD treatment: F _1, 21_ = 0.5369, *p* = 0.4722; Fig. 5F) and the KYN/TRP *1000 ratio (effect of morphine tolerance: F _1, 21_ = 23.74, *p* < 0.0001; effect of CBD treatment: F _1, 21_ = 1.583, *p* = 0.2228; interaction morphine tolerance ×CBD treatment: F _1, 21_ = 2.297, *p* = 0.1453; Fig. 5G) were significantly affected by morphine tolerance, with higher values observed in tolerant than in non-tolerant rats regardless of CBD treatment. The 5-HTP/TRP*1000 ratio (effect of morphine tolerance: F _1, 21_ = 7.905, *p* = 0.0111; effect of CBD treatment: F _1, 21_ = 9.012, *p* = 0.0073; interaction morphine tolerance ×CBD treatment: F _1, 21_ = 0.004, *p* = 0.9501; Fig. 5H) was significantly affected by morphine tolerance, with lower values observed in tolerant than in non-tolerant rats regardless of CBD treatment, and by CBD treatment, with the 5-HTP/TRP*1000 ratio significantlty increased by CBD compared to VEH treatment, reardelss of morphine tolerance (Fig. 5H).

Similar to TRP, DA levels (effect of morphine tolerance: F _1, 21_ = 7.060, *p* = 0.0147; effect of CBD treatment: F _1, 21_ = 0.7799, *p* = 0.3872; interaction morphine tolerance ×CBD treatment: F _1, 21_ = 7.060, *p* = 0.0147; Fig. 5I) were significantly higher in non-tolerant SNI rats treated with VEH compared to morphine-tolerant rats treated with either VEH or CBD.

### Brainstem

**Fig. 6:**
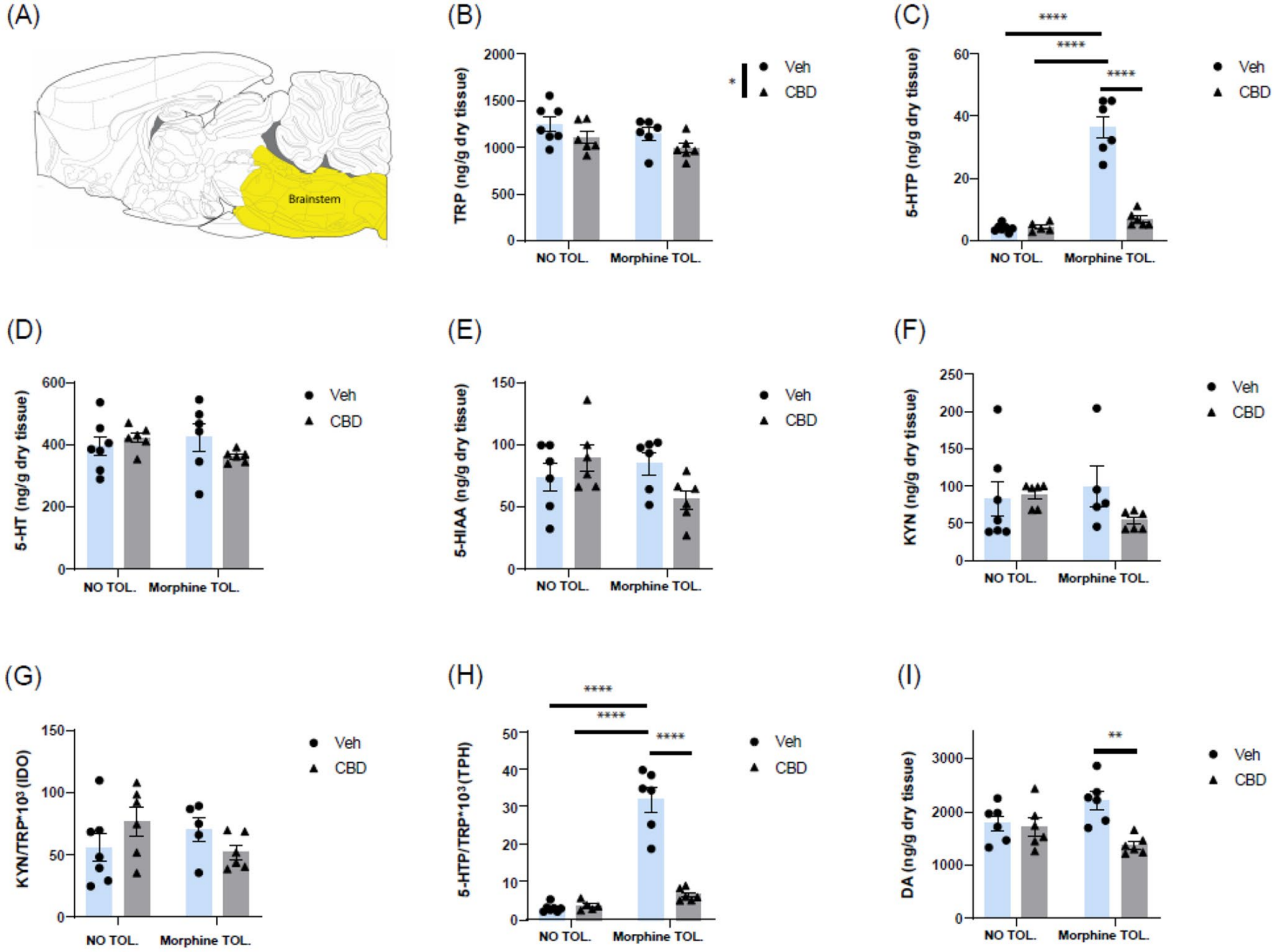
Effects of cannabidiol (CBD) treatment in sciatic nerve injured (SNI) rats tolerant (morphine TOL.) and non-tolerant (NO TOL.) to morphine on the metabolites of the tryptophan to serotonin and kynurenine pathways and dopamine in the brainstem: panels show (**a**) sagittal section of brainstem rat brain (coordinate: lateral 0.40 mm), from Paxinos and Watson [31], levels of (**b**) tryptophan (TRP), (**c**) 5-hydroxytryptophan (5-HTP), (**d**) 5-hydroxytryptamine (5-HT), (**e**) 5-hydroxyindole acetic acid (5-HIAA), (**f**) kynurenine (KYN), (**g**) 5-HTP/TRP*1000 ratio as a proxy of the activity of the enzyme tryptophan 5-hydroxylase (TPH), (**h**) KYN/TRP*1000 ratio as a proxy of the activity of the enzyme indoleamine 2,3-dioxygenase (IDO), and (**i**) dopamine (DA). Data are presented as mean ± SEM (*N* = 5–7 rats per group) with individual values indicated by dots (vehicle, veh) or triangles (CBD).**p < 0.01 and ****p < 0.0001, two-way ANOVA followed by Bonferroni post hoc test. # *p* < 0.05, main effect of morphine tolerance or CBD treatment following two-way ANOVA

The levels of various metabolites in the brainstem of SNI rats tolerant to morphine and treated with CBD are reported in Fig. 6.

CBD treatment significantly reduced TRP levels in the brainstem compared to VEH-treated animals (effect of morphine tolerance: F _1, 21_ = 2.632, *p* = 0.1197; effect of CBD treatment: F _1, 21_ = 4.719, *p* = 0.0414; interaction morphine tolerance ×CBD treatment: F _1, 21_ = 0.008, *p* = 0.9266; Fig. 6B). Morphine tolerance significantly increased the levels of 5-HTP compared to non-tolerant animals, whereas CBD treatment normalized 5-HTP levels in morphine-tolerant SNI rats to control levels. However, no significant effect of CBD was observed in non-tolerant rats (effect of morphine tolerance: F _1, 21_ = 84.00, *p* < 0.0001; effect of CBD treatment: F _1, 21_ = 58.54, *p* < 0.0001; interaction morphine tolerance ×CBD treatment: F _1, 21_ = 61.46, *p* < 0.0001; Fig. 6C). Neither morphine tolerance nor CBD significantly altered the levels of 5-HT (effect of morphine tolerance: F _1, 21_ = 0.2913, *p* = 0.5951; effect of CBD treatment: F _1, 21_ = 0.3269, *p* = 0.5736; interaction morphine tolerance ×CBD treatment: F _1, 21_ = 2–206, *p* = 0.1523; Fig. 6D), KYN (effect of morphine tolerance: F _1, 21_ = 0.2676, *p* = 0.6106; effect of CBD treatment: F _1, 21_ = 1.141, *p* = 0.2981; interaction morphine tolerance ×CBD treatment: F _1, 21_ = 1.937, *p* = 0.1793; Fig. 6F), or the KYN/TRP *1000 ratio (effect of morphine tolerance: F _1, 21_ = 0.2309, *p* = 0.6361; effect of CBD treatment: F _1, 21_ = 0.0185, *p* = 0.8931; interaction morphine tolerance ×CBD treatment: F _1, 21_ = 3.783, *p* = 0.0660; Fig. 6G). A significant interaction between morphine tolerance and CBD treatment (F _1, 21_ = 5.256, *p* = 0.0328) but no effects of either factors (effect of morphine tolerance: F _1, 21_ = 1.299, *p* = 0.2679; effect of CBD treatment: F _1, 21_ = 0.4785, *p* = 0.4970) was found for the levels of 5-HIAA (Fig. 6E).

An interaction between morphine tolerance and CBD treatment and an effects of both factors was observed on the 5-HTP/TRP*1000 ratio (effect of morphine tolerance: F _1, 21_ = 82.66, *p* < 0.0001; effect of CBD treatment: F _1, 21_ = 49.84, *p* < 0.0001; interaction morphine tolerance ×CBD treatment: F _1, 21_ = 55.16, *p* < 0.0001; Fig. 6H). Post-hoc omparisons showed that tolerance to morphine significantly increased the 5-HTP/TRP*1000 ratio, while CBD treatment reversed it to control levels in morphine-tolerant SNI rats. Regarding DA, CBD treatment significantly reduced DA levels in morphine-tolerant SNI rats (*p* = 0.0033) but had no effect in non-tolerant animals (effect of morphine tolerance: F _1, 21_ = 0.065, *p* = 0.8013; effect of CBD treatment: F _1, 21_ = 9.720 *p* = 0.005; interaction morphine tolerance ×CBD treatment: F _1, 21_ = 7.254, *p* = 0.0140; Fig. 6I).

### Serum

**Fig. 7:**
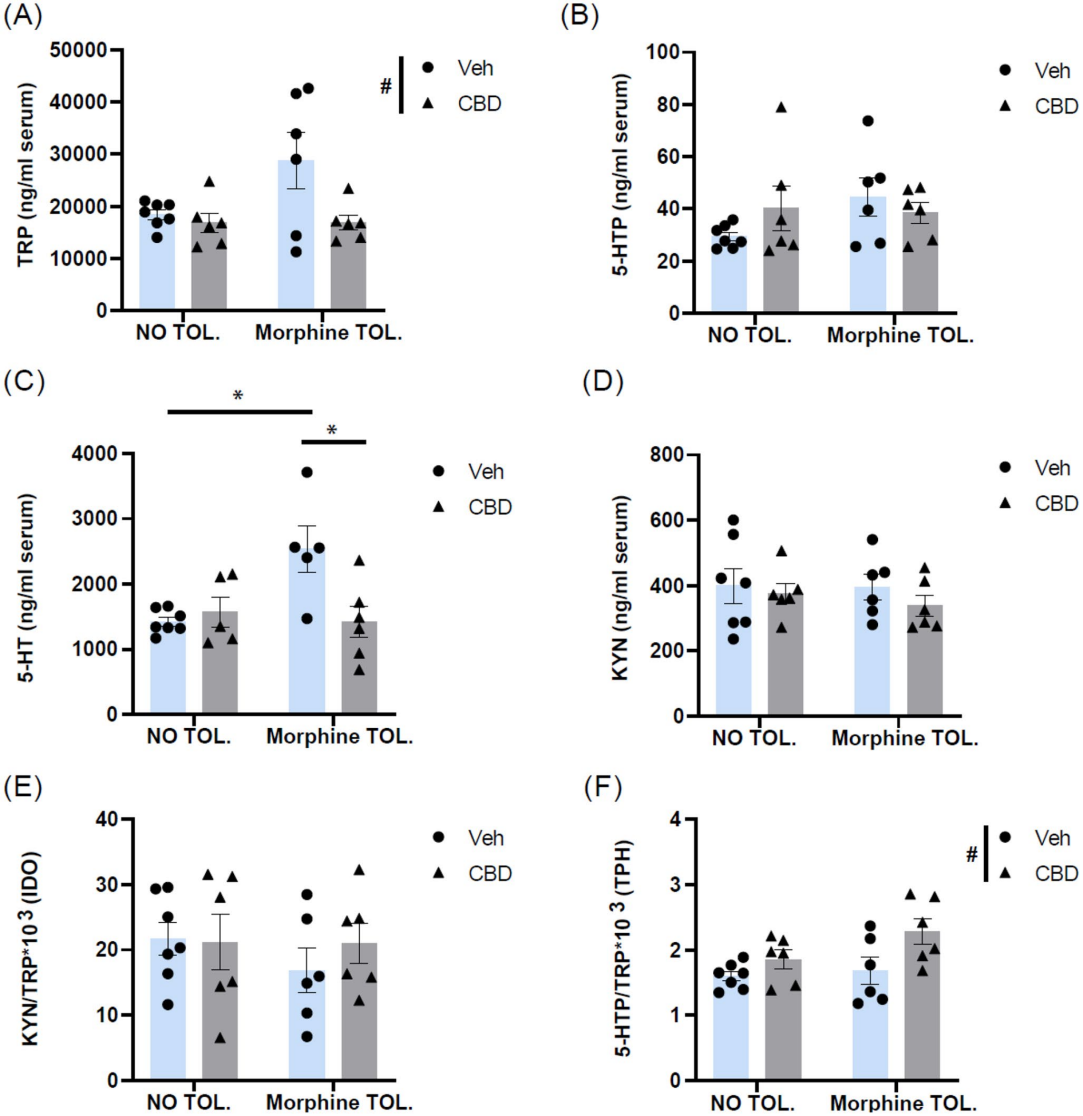
Effects of cannabidiol (CBD) treatment in sciatic nerve injured (SNI) rats tolerant (morphine TOL.) and non-tolerant (NO TOL.) to morphine on the metabolites of the tryptophan to serotonin and kynurenine pathways in the serum: panels show levels of (**a**) Tryptophan (TRP), (**b**) 5-hydroxytryptophan (5-HTP), (**c**) 5-hydroxytryptamine (5-HT), (**d**) Kynurenine (KYN), (**e**) Enzyme tryptophan hydroxylase (TPH) activity and (**f**) 5-HTP/TRP*1000 ratio as a proxy of the activity of the enzyme tryptophan 5-hydroxylase (TPH), and (**g**) KYN/TRP*1000 ratio as a proxy of the activity of the enzyme indoleamine 2,3-dioxygenase (IDO). Data are presented as mean ± SEM (*N* = 5–7 rats per group) with individual values indicated by dots (vehicle, veh) or triangles (CBD). *p < 0.05, two-way ANOVA followed by Bonferroni post hoc test. # *p* < 0.05, main effect of CBD treatment following two-way ANOVA

The levels of various metabolites in the serum of SNI rats tolerant to morphine and treated with CBD are reported in Fig. 7.

CBD treatment significantly reduced serum TRP levels, independent of morphine tolerance (effect of morphine tolerance: F _1, 21_ = 3.253, *p* = 0.0856; effect of CBD treatment: F _1, 21_ = 5.423, *p* = 0.0299; interaction morphine tolerance ×CBD treatment: F _1, 21_ = 3.135, *p* = 0.0912Fig. 7A). No significant effects of morphine tolerance or CBD treatment were observed on 5-HTP (effect of morphine tolerance: F _1, 21_ = 1.313, *p* = 0.2647; effect of CBD treatment: F _1, 21_ = 0.1660, *p* = 0.6878; interaction morphine tolerance ×CBD treatment: F _1, 21_ = 2.159, *p* = 0.1565; Fig. 7B) and KYN levels (effect of morphine tolerance: F _1, 21_ = 0.2670, *p* = 0.6108; effect of CBD treatment: F _1, 21_ = 0.9434, *p* = 0.3424; interaction morphine tolerance ×CBD treatment: F _1, 21_ = 0.1671, *p* = 0.6868; Fig. 7D), or the KYN/TRP *1000 ratio (effect of morphine tolerance: F _1, 21_ = 0.5578, *p* = 0.4634; effect of CBD treatment: F _1, 21_ = 0.3014, *p* = 0.5888; interaction morphine tolerance ×CBD treatment: F _1, 21_ = 0.4872, *p* = 0.4929; Fig. 7E). Serum levels of 5-HT were significantly higher in morphine-tolerant rats compared to non-tolerant animals. However, CBD treatment reduced 5-HT levels in morphine-tolerant rats to levels comparable to non-tolerant animals (effect of morphine tolerance: F _1, 21_ = 4.437, *p* = 0.0487; effect of CBD treatment: F _1, 21_ = 4.522, *p* = 0.0468; interaction morphine tolerance ×CBD treatment: F _1, 21_ = 7.751, *p* = 0.0118; Fig. 7C). Finally, the 5-HTP/TRP*1000 ratio was significantly increased by CBD treatment, regardless of morphine tolerance status (effect of morphine tolerance: F _1, 21_ = 2.595, *p* = 0.1221; effect of CBD treatment: F _1, 21_ = 7.249, *p* = 0.0136; interaction morphine tolerance ×CBD treatment: F _1, 21_ = 1.181, *p* = 0.2895; Fig. 7F).

## Discussion

**Fig. 8:**
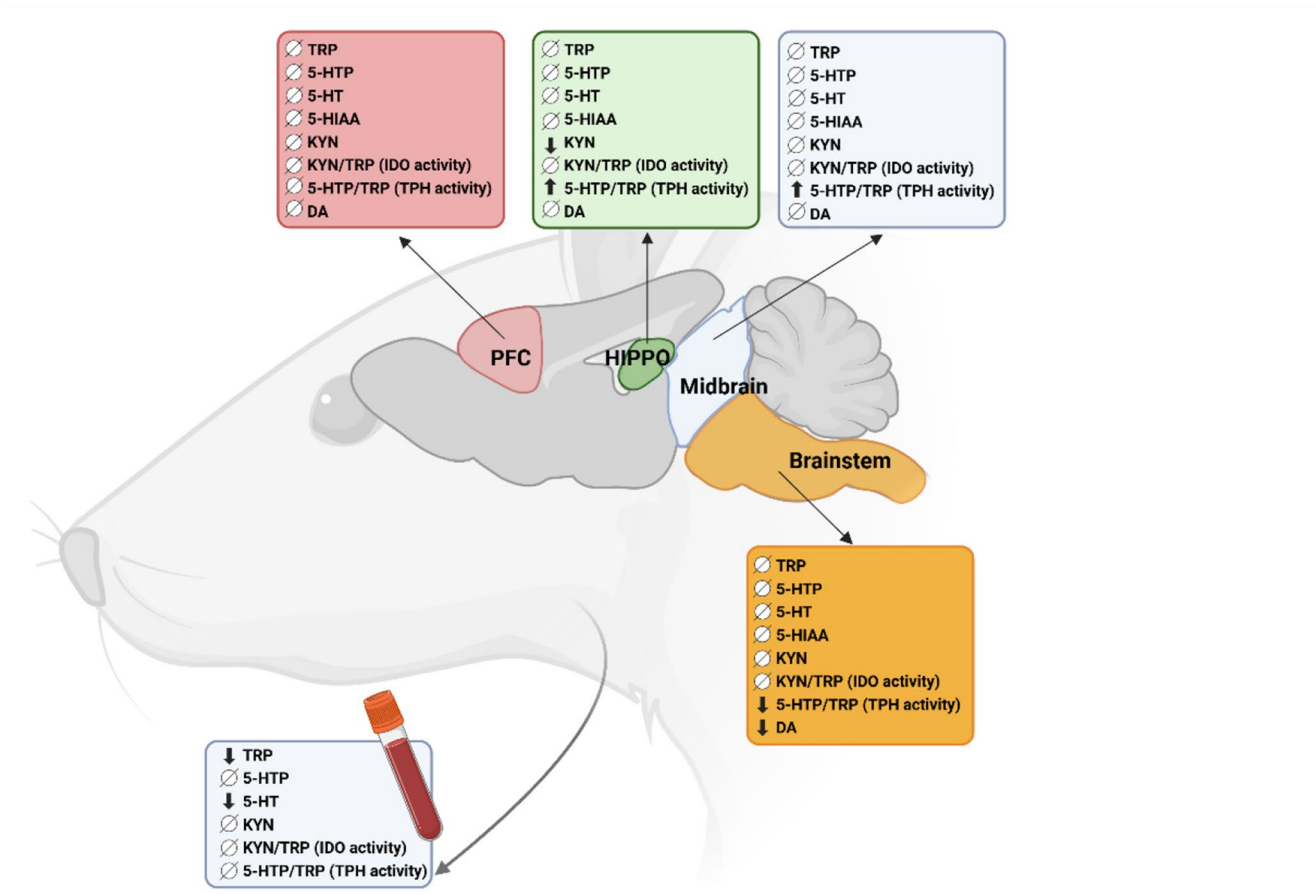
Summary of neurochemical changes induced by CBD in morphine-tolerant rats across different brain regions and serum. This schematic summarizes the direction of significant changes in tryptophan metabolism and dopamine (DA) levels in morphine-tolerant SNI rats following CBD treatment (20 mg/kg, ip). Arrows indicate the direction of significant effects observed in each region: ↓ = decrease; ↑ = increase; ⦰ = no significant change. Tryptophan (TRP), 5-hydroxytryptophan (5-HTP), serotonin (5-HT), its metabolite 5-hydroxyindoleacetic acid (5-HIAA), kynurenine (KYN), and dopamine (DA). TPH, tryptophan hydroxylase; IDO, indoleamine 2,3-dioxygenase. HIPPO, hippocampus; PFC, prefrontal cortex

This study investigated the effects of a single injection of CBD on TRP metabolism and DA in a rat model of NP tolerant to morphine. Chronic morphine administration led to a progressive decrease in the PWT, confirming the development of tolerance. Notably, behavioral data revealed a significant effect of CBD (20 mg/kg) on mechanical hypersensitivity independent of the development of morphine tolerance. However, it is important to note that when CBD was administered to rats with established morphine tolerance, it exerted a modest effect on mechanical hypersensitivity, without fully restoring the antinociceptive threshold to non-tolerant levels. This partial behavioral efficacy parallels our neurochemical findings showing that CBD did not induce a global reversal of all morphine-induced metabolic alterations but a selective modulation of TRP metabolism and DA within specific key regions involved in pain. Indeed, CBD significantly modulated TRP metabolism in morphine-tolerant animals (see Fig. 8 for a summary of main findings), as evidenced by an increased 5-HTP/TRP ratio in the hippocampus and midbrain and reduced KYN levels in the hippocampus, suggesting a metabolic shift toward 5-HT synthesis. Moreover, CBD decreased DA levels in the brainstem and altered peripheral TRP metabolism, reducing both TRP and 5-HT concentrations and lowering the 5-HTP/TRP ratio in serum. These findings indicate that while a single injection of CBD primarily alleviates mechanical hypersensitivity in the absence of morphine tolerance, its antinociceptive effects under tolerant conditions remain limited despite clear neurochemical actions on TRP metabolism and DA. Crucially, these data highlight a dissociation between neurochemical modulation and behavioral recovery in the morphine tolerant state. It is important to note that neurochemical assessments were performed 6 hours post-administration, a time point when the acute effects had largely diminished. Therefore, the observed neurochemical profile reflects long-term metabolic modulation rather than the real-time neurochemical correlates of acute analgesia. These differential effects likely indicate that localized and sustained modulation of TRP metabolism and partial correction of morphine-induced neurochemical imbalances may not be sufficient to overcome the functional analgesic deficits associated with opioid tolerance. Specifically, CBD promotes the conversion of TRP into 5-HT rather than KYN, particularly in the midbrain and hippocampus, likely via activation of TPH, the rate-limiting enzyme in 5-HT biosynthesis. It should be noted that in some brain regions and for several TRP metabolites, as well as for the CBD behavioural effect, there is an absence of a significant morphine tolerance ×CBD treatment interaction. Consequently, this has limited the ability to statistically differentiate the effects of CBD in morphine tolerant versus non-tolerant SNI rats. From a biological perspective, this lack of interaction suggests that the observed neurochemical effects of morphine tolerance and acute CBD treatment likely occur through additive or parallel independent mechanisms rather than synergistic or antagonistic ones.

### Morphine tolerance, CBD, and serotonergic pathway modulation

TRP metabolism plays a central role in both 5-HT synthesis and KYN pathway activation, two interconnected systems critically involved in the modulation of pain and neuroinflammatory processes [32]. The descending pain modulatory pathway, including the periaqueductal gray (PAG), rostroventral medulla (RVM), and PFC, is essential for opioid-induced analgesia [33–37]. In addition, the hippocampus, traditionally known for its role in learning and memory, is now recognized as an important structure in pain modulation, especially chronic pain [38, 39]. It receives nociceptive input through both direct spinal projections and indirect ascending pathways, allowing it to participate in the integration of sensory, cognitive, and emotional aspects of the pain experience. Structural and functional alterations of the hippocampus, such as reduced volume and disrupted connectivity, have been consistently observed in individuals with chronic pain [39]. These changes are often accompanied by cognitive impairments and affective symptoms, including memory deficits, anxiety, and depression. Furthermore, variations in hippocampal integrity appear to influence pain sensitivity, and emerging evidence suggests that distinct hippocampal subregions may play differential roles in pain processing [39, 40]. Morphine tolerance was associated with altered TRP metabolism, particularly in the midbrain and PFC. Indeed, morphine-tolerant rats exhibited significantly lower levels of TRP and its metabolites along the 5-HT pathway, including 5-HTP, 5-HT, and 5-hydroxyindoleacetic acid (5-HIAA), in the midbrain, suggesting impaired TRP availability and metabolism along the 5-HT function, potentially weakening endogenous pain modulation. The mechanisms underlying morphine tolerance involve complex neuroadaptive changes, including the β-arrestin pathway. Indeed, chronic morphine exposure promotes β-arrestin recruitment to the mu opioid receptor (MOR), leading to MOR desensitization and internalization [41]. Our findings suggest that the altered TRP metabolism in morphine tolerance could be a downstream consequence of β-arrestin-mediated dysregulation [42]. The effect on mechanical hypersensitivity observed after a single dose of CBD (20 mg/kg), although of limited magnitude in morphine-tolerant animals, suggests that CBD may influence opioid receptor responsiveness, potentially through mechanisms involving β-arrestin-mediated signaling and receptor desensitization. This highlights the need for further studies to dissect the interaction between CBD and intracellular pathways regulating opioid tolerance. Interestingly, we observed that CBD treatment significantly increased the 5-HTP/TRP ratio in the hippocampus and midbrain, suggesting enhanced TPH activity, the rate-limiting enzyme in 5-HT synthesis [43]. This aligns with our previous findings showing that CBD modulates 5-HT neuronal activity in the dorsal raphe nucleus [25]. However, this enzymatic enhancement did not translate into a widespread restoration of 5-HT tone. Indeed, while CBD increased the 5-HTP/TRP ratio, suggesting enhanced 5-HT synthesis capacity, this was not accompanied by a robust increase in 5-HT levels in all regions. Furthermore, critical regions for pain modulation, such as the PFC, did not show the same responsiveness to CBD in tolerant animals as observed in non-tolerant controls. This inability of CBD to engage the PFC serotonergic system in morphine-tolerant rats may partially explain the lack of robust antinociception. Overall, these findings suggest that while CBD modulates TRP metabolism, its effects on overall 5-HT availability are complex and may involve region-specific mechanisms or altered reuptake/metabolism downstream of synthesis. Additionally, it is worth mentioning that CBD may indirectly modulate 5-HT by IDO [12, 44], a key enzyme in the KYN pathway that competes for TRP availability for the synthesis of 5-HT.

### Influence of CBD on the KYN pathway and neuroinflammation

Morphine tolerance was associated with significant alterations in the conversion of TRP into KYN, with morphine-tolerant rats displaying decreased KYN levels in the PFC and hippocampus, suggesting dysregulated TRP metabolism via IDO and TDO. Notably, CBD treatment significantly reduced hippocampal KYN levels, supporting the hypothesis that CBD modulates IDO activity [44]. Interestingly, the reduction of KYN in the hippocampus occurred despite no changes in the KYN/TRP ratio, suggesting localized inhibition of IDO activity or enhanced KYN clearance. Although systemic IDO activity, as reflected by the KYN/TRP ratio, was unchanged, the reduction in KYN suggests localized modulation of inflammatory pathways by CBD. Given that neurotoxic KYN metabolites, particularly quinolinic acid, contribute to NP via excitotoxic mechanisms [45], the ability of CBD to lower KYN levels while increasing 5-HT activity [25] may be relevant to its mechanism of action. These findings reinforce the role of KYN pathway dysregulation in NP [46] and suggest that the therapeutic potential of CBD lies more in modulating neuroinflammation [47] than in direct pain relief [48, 49] in the context of opioid tolerance.

### Dopaminergic alterations in morphine tolerance and CBD effects

The DA system, in contrast, plays a crucial role in reward processing and opioid-induced plasticity, with dysregulation contributing to opioid tolerance and dependence [50, 51]. The ascending spinothalamic pathway, which includes the midbrain, medulla oblongata, and spinal cord, integrates pain signals and is influenced by dopaminergic transmission [52, 53]. DA plays a crucial role in pain modulation [54, 55] and reward processing [56, 57]. Our results indicate that morphine tolerance is associated with reduced DA levels in the PFC and midbrain, consistent with previous findings on chronic opioid exposure disrupting DA signaling, finally contributing to opioid-induced hyperalgesia and addiction-related behaviors [50]. Interestingly, CBD treatment did not affect DA levels in the PFC or midbrain in morphine-tolerant rats. Additionally, it significantly reduced DA levels in the brainstem, an effect uniquely observed in the morphine-tolerant group. This suggests that CBD exerts region-specific effects on DA neurotransmission. Previous research indicates that CBD may interact with DA signaling via CB1, CB2, and TRPV1 receptors [58, 59] and through modulation of neuroinflammation [60, 61]. The observed decrease in brainstem DA levels following CBD treatment raises the possibility that CBD modulates opioid-induced DA dysregulation [62, 63], though whether this reduction is beneficial or maladaptive remains to be elucidated.

### Peripheral TRP and 5-HT modulation by CBD

Serum analysis revealed that morphine-tolerant rats exhibited elevated peripheral levels of 5-HT, which were effectively reversed by CBD treatment [25, 64]. This may suggest that chronic morphine exposure enhances systemic 5-HT production, potentially as a compensatory response to central 5-HT deficits. Consequently, the ability of CBD to modulate peripheral 5-HT homeostasis raises important questions regarding the cross-talk between systemic TRP metabolism [65] and central serotonergic signaling. While these findings might reflect distinct regional metabolic regulations or alterations in amino acid transport kinetics across the blood-brain barrier, further investigations are required to verify the specific dynamics driving these differences in the morphine-tolerant state.

### Limitations and future directions

While our findings provide new insights into the neurochemical effects of CBD on morphine tolerance, several limitations should be considered. We have not included a non-injured (naïve or sham) control group. Consequently, our conclusions are strictly limited to the comparison between morphine-tolerant and non-tolerant states within the neuropathic phenotype. In our protocol, CBD was administered acutely at a single dose (20 mg/kg), and tissues were collected at 6 hours post-administration. This time point allowed us to assess the lasting metabolic impact of CBD but implies that the neurochemical data reflect a state where the acute behavioral antinociception had already subsided. Consequently, the observed changes should be interpreted as long-term modulatory effects rather than the direct drivers of acute pain relief. Future studies should include time-course and dose–response assessments as well as repeated-dose paradigms to better determine the therapeutic potential of CBD in morphine-tolerant individuals and to correlate peak behavioral effects with simultaneous neurochemical changes. Additionally, the observed region-specific alterations in neurotransmitter metabolism suggest complex and localized effects of CBD. Further molecular and mechanistic studies are necessary to delineate how CBD influences TRP metabolism via KYN and 5-HT, and DA pathways at both the enzymatic and receptor levels. Sex differences represent another limitation, as opioid tolerance and NP [66, 67], as well as the effects of CBD [68], which seems to be dependent on ovarian hormones [69], and tryptophan metabolism [70] exhibit well-documented sex-dependent variations. Future studies should also include females to enhance translational relevance and determine whether the effects of CBD on opioid tolerance and neurochemical pathways differ across sexes. Finally, we did not study TRP metabolites and DA in spinal cord regions such as the lumbar dorsal horn, although they are also central in NP processing, and measure directly inflammatory cytokines and immune markers, which would have provided a more detailed understanding of the role of CBD in modulating neuroinflammation and the KYN pathway in morphine tolerance.

## Conclusion

Our study provides evidence that acute CBD may have limited antinociceptive efficacy following chronic morphine treatment and the development of tolerance in NP. This opioid-induced tolerance also alters TRP metabolism via both 5-HT and KYN pathways, with significant reductions in 5-HT and DA activities observed in key brain regions modulating pain. Additionally, we have demonstrated that in a brain region-dependent manner, CBD influences the metabolism of TRP into 5-HT by enhancing TPH activity, especially at the level of the midbrain and hippocampus. However, this metabolic shift observed at 6 hours post-administration did not translate into sustained effects on mechanical hypersensitivity in morphine-tolerant rats. This discrepancy underscores that long-term metabolic modulation does not equate to functional restoration. CBD influenced not only the 5-HT synthesis, but also neuroinflammatory markers (IDO activity and KYN levels) while alleviating some mechanical hypersensitivity in morphine-tolerant rats, suggesting a dual action on pain perception and neurochemical balance. The potential interplay between the TRP *via* 5-HT and KYN pathways and the DA system suggests that the effects of CBD on morphine tolerance could be mediated by an integrated modulation of pain-related neurotransmitter networks rather than isolated pathways. Future studies should aim to clarify the functional relationship between TRP-derived metabolites and DA activity in the context of CBD treatment. Overall, these findings further highlight the complex pharmacology of CBD and underscore the need for further research on its therapeutic potential in opioid tolerance and NP management.

## Data Availability

Data is available on request.

## Abbreviations

5-HIAA: 5-Hydroxyindoleacetic acid
5-HT: 5-Hydroxytryptamine (serotonin)
5-HTP: 5-Hydroxytryptophan
AUC: Area under the curve
CBD: Cannabidiol
CB1 / CB2: Cannabinoid receptor type 1 / type 2
CNS: Central nervous system
DA: Dopamine
D1 / D7: Day 1 / Day 7 (of morphine treatment)
EtOH: Ethanol
HPLC: High-performance liquid chromatography
IDO / IDO1 / IDO2: Indoleamine 2,3-dioxygenase (isoforms 1 and 2)
IFN-γ: Interferon-gamma
Ip: Intraperitoneal
KYN: Kynurenine
MOR: Mu opioid receptor
NP: Neuropathic pain
PAG: Periaqueductal gray
PFC: Prefrontal cortex
PWT: Paw withdrawal threshold
RVM: Rostroventral medulla
sc: Subcutaneous
SEM: Standard error of the mean
SNI: Spared nerve injury
TPH: Tryptophan hydroxylase
TDO: Tryptophan 2,3-dioxygenase
TNF-α: Tumor necrosis factor-alpha
TRP: Tryptophan
UV-VIS: Ultraviolet–visible (detector)
VEH: Vehicle
VTA: Ventral tegmental area
